# Phenyl-Bis-Naphthyl Derivative-Based Artificial Light-Harvesting System for Singlet Oxygen Oxidation

**DOI:** 10.3390/molecules30224424

**Published:** 2025-11-16

**Authors:** Liangtao Pu, Yonglei Chen, Guangping Sun

**Affiliations:** 1 School of Urban Construction, Changzhou University, Changzhou 213164, China; 2 School of Chemistry and Chemical Engineering, Nantong University, Nantong 226019, China

**Keywords:** artificial light-harvesting, host–guest interaction, supramolecular assembly, energy transfer, singlet oxygen oxidation

## Abstract

A novel artificial light-harvesting system (LHS) for the photooxidation reaction was constructed by the phenyl-bis-naphthyl derivative (**PBN**) and water-soluble phosphate-pillar[5]arene (**WPP5**). After host–guest interaction, **WPP5** integrated with **PBN** to form **WPP5-PBN** amphiphiles, which self-assembled to **WPP5-PBN** nanoparticles. Based on the aggregate state of **PBN** in **WPP5-PBN** nanoparticles, **WPP5-PBN** nanoparticles emitted a significant yellow fluorescence as energy donors. Due to the yellow fluorescence fully covering the absorption of sulforhodamine 101 (**SR101**), **SR101** was used as energy acceptors and loaded in **WPP5-PBN** nanoparticles for constructing the **WPP5-PBN-SR101** LHS, whose energy transfer efficiency and antenna effect were 66.32% and 22.34. Notably, after the energy of the **WPP5-PBN** antenna transferred to **SR101**, more singlet oxygen (^1^O_2_) production was observed in the **WPP5-PBN-SR101** LHS, which was successfully used as a photocatalyst to catalyze the oxidation reaction of 4-methoxythioanisole to 1-methoxy-4-(methylsulfinyl)benzene, imitating the solar energy conversion to chemical energy.

## 1. Introduction

Photosynthesis is one of the most important light-harvesting and energy transfer processes in nature, which converts solar energy to chemical energy for life activities [1,2]. In a natural light-harvesting system (LHS), solar energy is absorbed by a pigment-protein complex and transferred to the reaction center for photocatalysis, which achieve solar energy conversion and storage [3,4]. Currently, inspired by the solar energy utilization of natural LHS, more artificial light-harvesting systems are being developed to imitate the solar energy transfer and conversion behavior by fluorescence resonance energy transfer (FRET) [5,6,7,8]. Initially, artificial LHSs were usually constructed by aggregation-caused quenching (ACQ) donors, which underwent multi-step covalent synthesis in an organic environment and performed lower emission efficiency in an aqueous environment. Fortunately, Tang et al. revealed a novel emission mechanism of aggregation-induced emission (AIE) in an aggregate state, which provided an approach to designing aggregate state donors and constructing artificial LHSs in an aqueous environment [9,10]. Recently, supramolecular strategy has been widely used in the construction of AIE-based artificial LHSs in an aqueous environment because of the higher assembly efficiency and the lower synthesis limit [11,12]. Moreover, supramolecular AIE-based artificial LHSs can not only actualize the energy transfer in an aqueous environment, but also finish the solar energy output for photocatalysis [13,14,15]. For example, Hu et al. reported a tetraphenylethylene-embedded pillar[5]arene-based artificial LHS, which significantly achieved the dehalogenation of bromoacetophenone in an aqueous environment [13]. Yi et al. developed a tetraphenylethylene-based artificial light-harvesting metallacycle system, which was successfully utilized to catalyze the C-H alkylation reaction in an aqueous solution [14]. Xing et al. designed a triphenylamine-based supramolecular artificial light-harvesting organic framework, which exhibited the significant photocatalytic ability of C-P bond coupling [15]. However, the photocatalysis applications of artificial LHSs always focused on the traditional chemical reactions and photocatalytic oxidation to sulfoxide was rarely reported. Therefore, it was meaningful to design a novel artificial LHS for the photocatalytic oxidation reaction in water.

**Scheme 1:**
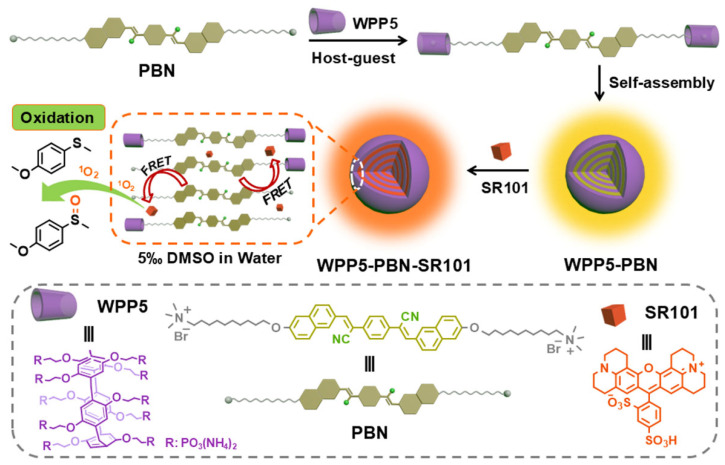
Schematic illustration of phenyl-bis-naphthyl derivative-based artificial light-harvesting system for singlet oxygen oxidation.

Herein, a novel artificial LHS for the photooxidation reaction to sulfoxide was constructed by the host–guest interaction of phenyl-bis-naphthyl derivative (**PBN**) and water-soluble phosphate-pillar[5]arene (**WPP5**). As shown in Scheme 1, after **WPP5** binding with **PBN**, the supramolecular amphiphilic **WPP5-PBN** self-assembled to **WPP5-PBN** nanoparticles, which emitted significant yellow fluorescence as donors. Due to the yellow emission of **WPP5-PBN** nanoparticles fully covering the absorption of sulforhodamine 101 (**SR101**), **SR101** was loaded as acceptors in **WPP5-PBN** nanoparticles. After tightly stacking in an assembly process, a prominent fluorescence resonance energy transfer (FRET) was constructed in **WPP5-PBN-SR101** nanoparticles and the harvested energy of **WPP5-PBN** antennas was transferred to **SR101**, whose fluorescence significantly changed to a red emission, confirming the construction of **WPP5-PBN-SR101** LHS. Moreover, benefitting from the energy transfer behavior, more energy was transferred to excite **SR101**, which achieved a significant singlet oxygen (^1^O_2_) production. Notably, ^1^O_2_ could be used as photocatalysts to catalyze the oxidation reaction of 4-methoxythioanisole to 1-methoxy-4-(methylsulfinyl)benzene, which successfully imitated the energy conversion of the solar energy to chemical energy, suggesting **WPP5-PBN-SR101** potential in artificial light-harvesting energy storage.

## 2. Results and Discussion

### 2.1. Construction of Supramolecular Nanoparticles

**Scheme 2:**
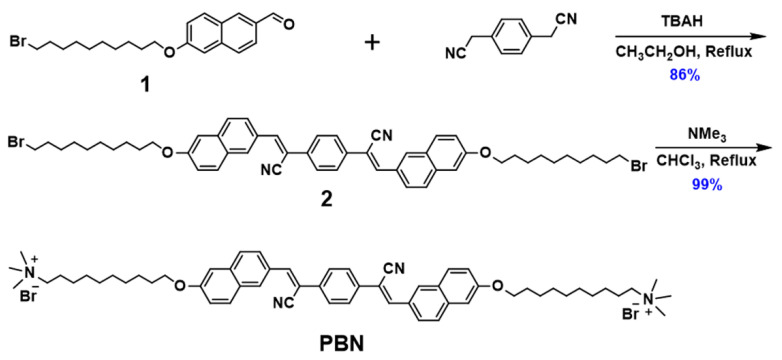
Synthesis route of **PBN**.

**Figure 1:**
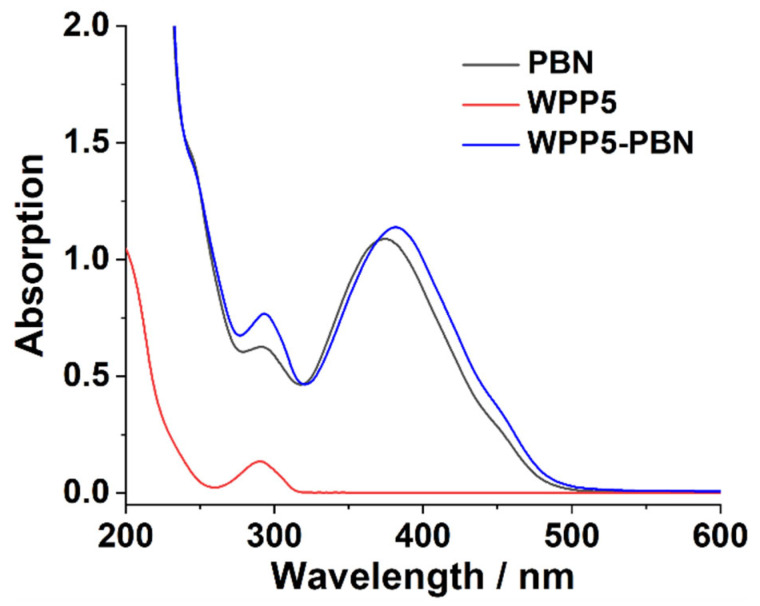
The host–guest interaction absorption spectra of **WPP5** and **PBN** in water: [**PBN**] = 50.00 μM, [**WPP5**] = 7.50 μM, and [**PBN**]:[**WPP5**] = 20:3.

**WPP5** was synthesized according to the reported procedures [16,17]. **PBN** was synthesized by using the 6-hydroxy-2-naphthaldehyde as starting materials, which suffered three-step reactions (Scheme 2 and Figures S1–S7). After obtaining **WPP5** and **PBN**, their host–guest interaction was investigated by UV–Vis absorption spectrum [4,16]. As shown in Figure 1, the 320–500 nm absorption peak of **PBN** appeared a significant red shift after mixing with the **WPP5** solution, confirming their host–guest interaction in water.

**Figure 2:**
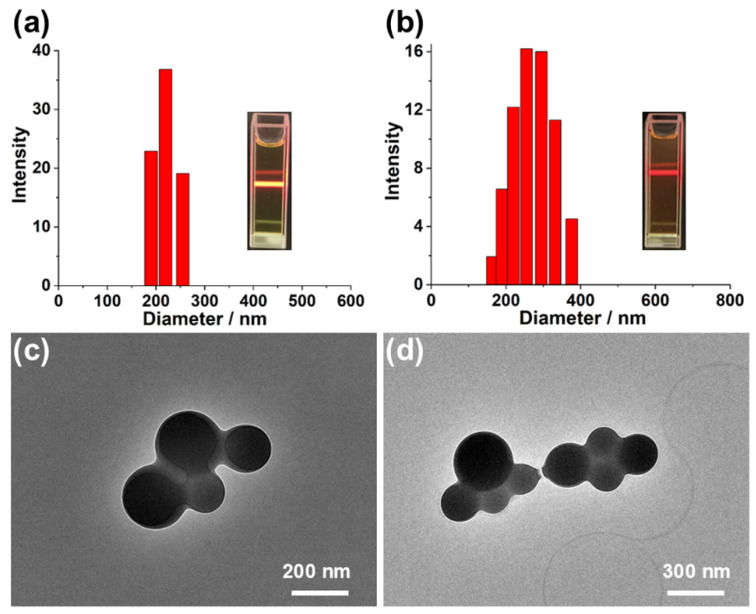
DLS distributions of (**a**) **WPP5-PBN** and (**b**) **WPP5-PBN-SR101** nanoparticles. TEM images of (**c**) **WPP5-PBN** and (**d**) **WPP5-PBN-SR101** nanoparticles. Inset Tyndall effect photos of **WPP5-PBN** and **WPP5-PBN-SR101**: [**PBN**] = 50.00 μM, [**WPP5**] = 7.50 μM, [**SR101**] = 0.50 μM, and [**PBN**]:[**WPP5**]:[**SR101**] = 100:15:1.

Moreover, after adding **WPP5** to **PBN** solution for a while, a significant Tyndall effect light-path was generated in the **WPP5-PBN** solution by laser pen irradiation, revealing the formation of **WPP5-PBN** nanoparticles (Figure 2a). Fortunately, benefitting from the formation of **WPP5-PBN** nanoparticles, a special space structure was generated in the **WPP5-PBN** nanoparticle. Due to the rich C-H and π bonds in the chemical structures of **PBN**, **WPP5**, and **SR101**, when **SR101** was added to the solution of **WPP5** and **PBN**, **SR101** could effectively load in the layers of **WPP5-PBN** via C-H···π and π···π stacking interactions [18,19]. For further exploring the details of these supramolecular nanoparticles, their nano-size distribution and micromorphology were investigated by dynamic light scattering (DLS) and transmission electron microscope (TEM). As shown in Figure 2, the DLS result of **WPP5-PBN** nanoparticles displayed a small-scale distribution of 190–260 nm, whose average diameter was around 200 nm. The TEM image indicated **WPP5-PBN** nanoparticles to have a sphere shape. After loading **SR101**, the distribution of **WPP5-PBN-SR101** nanoparticles significantly changed to 160–380 nm, but the morphology of **WPP5-PBN-SR101** nanoparticles also exhibited a spherical shape (Figure 2b,d). Furthermore, the nanostructure of **WPP5-PBN-SR101** was investigated by X-ray photoelectron spectroscopy (XPS). As shown in Figure S8, the significant C-S, S=O, and S-O signals of **SR101** were observed, confirming the encapsulation of **SR101** in **WPP5-PBN-SR101** nanoparticles.

### 2.2. Investigation of Energy Transfer Process

**Figure 3:**
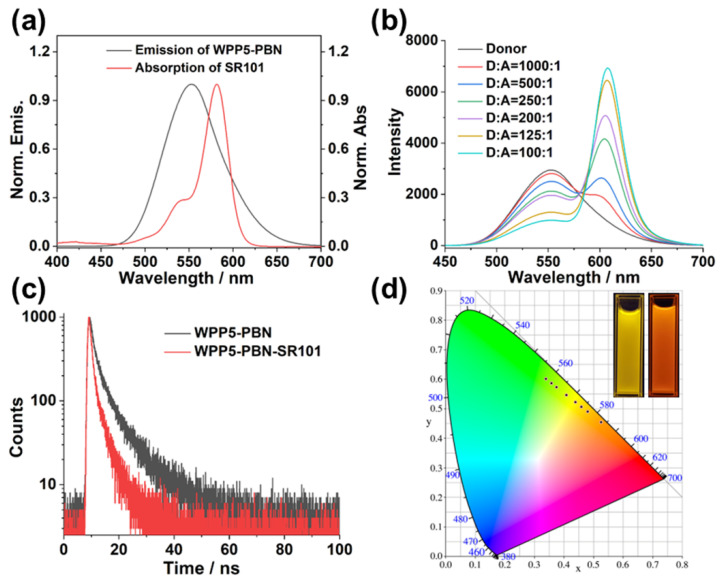
(**a**) Emission of **WPP5-PBN** and absorption of **SR101**: [**PBN**] = 50.00 μM, [**WPP5**] = 7.50 μM, and [**SR101**] = 50.00 μM. (**b**) Fluorescence spectra of energy transfer in **WPP5-PBN-SR101** from 1000:1 to 100:1 (D:A): [**PBN**] = 50.00 μM, [**WPP5**] = 7.50 μM, and [**SR101**] = 0.05 (1000:1), 0.10 (500:1), 0.20 (250:1), 0.25 (200:1), 0.40 (125:1), 0.50 (100:1) μM. (**c**) Lifetime curve spectra of **WPP5-PBN** and **WPP5-PBN-SR101**. The excitation wavelength was 365 nm and the observation wavelength was 551 nm. (**d**) CIE diagram of energy transfer process in **WPP5-PBN-SR101**. Inset fluorescence photos of **WPP5-PBN** (left) and **WPP5-PBN-SR101** (right).

Based on the AIE feature of **PBN** guest, the aggregated **PBN** in **WPP5-PBN** nanoparticles emitted significant yellow fluorescence, which could conduct as energy donors (D) to construct an energy transfer process. As shown in Figure 3a, as **WPP5-PBN** nanoparticles’ yellow fluorescence entirely covered the absorption of **SR101**, **SR101** was considered as energy acceptors (A) to receive the energy from the **WPP5-PBN** artificial light-harvesting antenna. After loading **SR101** in **WPP5-PBN** nanoparticles, **SR101** was tightly loaded in **WPP5-PBN** amphiphiles and the donor–acceptor distance was in the ideal state, arising a significant FRET process in **WPP5-PBN-SR101** nanoparticles. With the molar ratio of **SR101** loaded from 1000:1 to 100:1 (D:A), **WPP5-PBN**’s emission at 551 nm gradually decreased, but the characteristic emission of **SR101** at 608 nm exhibited a rapid increase, displaying an efficient artificial energy transfer process in **WPP5-PBN-SR101** nanoparticles (Figure 3b). In addition, according to the reported investigation in the energy transfer process [4,8], fluorescence color changes were significant signals for confirming the energy transfer process. The fluorescence spectra of the energy transfer process were often converted into fluorescence color coordinate points in a CIE chromaticity diagram, which clearly revealed the fluorescence color change trajectory, identifying the energy transfer process. Thus, when the fluorescence spectra of the above energy transfer process were converted into fluorescence color coordinate points in a CIE chromaticity diagram, the coordinate points in the CIE chromaticity diagram indicated varying amounts of additive and the energy transfer process was clearly observed according to the coordinate trajectory from yellow to red regions, corresponding to the fluorescence color changes from yellow emission in the **WPP5-PBN** solution to red emission in the **WPP5-PBN-SR101** solution (Figure 3d). Moreover, the artificial light-harvesting behaviors were also verified by tracking the lifetime changes in donors (**WPP5-PBN**) in the energy transfer process [4,8,16]. According to the time-correlated single photon counting (TCSPC) curve of **WPP5-PBN** nanoparticles, the τ_1_ and τ_2_ of donors were determined to be 2.19 ns and 9.34 ns by the bis-exponential fitting calculation (Figure S9). However, after loading **SR101** in **WPP5-PBN** nanoparticles, the τ_1_ and τ_2_ of the donors in **WPP5-PBN-SR101** nanoparticles were decreased to 0.96 ns and 4.69 ns, attributing to **WPP5-PBN**’s energy transferred to **SR101** (acceptors) (Figure S10). The τ of the donor was significantly changed from 6.48 ns in **WPP5-PBN** nanoparticles to 2.64 ns in **WPP5-PBN-SR101** nanoparticles, suggesting a convincing energy transfer process in **WPP5-PBN-SR101** nanoparticles. The energy transfer efficiency and antenna effect were calculated to be 66.32% and 22.34 in **WPP5-PBN-SR101** nanoparticles, displaying the significant light-harvesting ability for energy transfer utilization (Figures S11 and S12).

### 2.3. Investigation of Photooxidation Reaction

**Figure 4:**
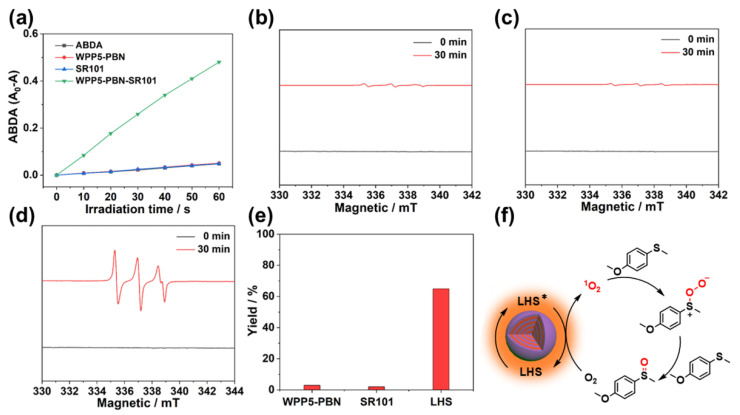
(**a**) Absorbance quench spectra of ABDA, **WPP5-PBN**-ABDA, **SR101**-ABDA, and **WPP5-PBN-SR101**-ABDA solution at 376 nm. EPR spectra of (**b**) **WPP5-PBN**, (**c**) **SR101**, and (**d**) **WPP5-PBN-SR101** solution. (**e**) The photooxidation yields of **WPP5-PBN**, **SR101** and **WPP5-PBN-SR101** LHS. (**f**) The proposed ^1^O_2_ oxidation mechanism. * indicate the excited state.

Subsequently, the **WPP5-PBN-SR101** LHS was used as a photocatalyst to investigate the harvested energy output for utilization. As shown in Figure S13, 9,10-anthracenediylbis(methylene)dimalonic acid (ABDA) was applied as a singlet oxygen (^1^O_2_) indicator to explore the ^1^O_2_ production ability in the **WPP5-PBN-SR101** LHS according to the absorbance quench at 376 nm [20]. No significant absorbance quench was observed in **WPP5-PBN** and **SR101** groups, displaying **WPP5-PBN** and **SR101** to have weak ^1^O_2_ production ability (Figure 4a). But, after the light-harvesting process in **WPP5-PBN-SR101**, a significant absorbance quench at 376 nm was found, which displayed more ^1^O_2_ produced in the **WPP5-PBN-SR101** LHS due to more energy of **WPP5-PBN** (donors) transferred to excite **SR101** (Figure 4a and Figure S14). In addition, the ^1^O_2_ production ability of the **WPP5-PBN-SR101** LHS was directly measured by electron paramagnetic resonance (EPR), which used 2,2,6,6-tetramethylpiperidine (TEMP) as the ^1^O_2_ trapping agent. As shown in Figure 4b,c, the ^1^O_2_ signals were weak in **WPP5-PBN** and **SR101** groups under 365 nm irradiation for 30 min, in accordance with their ABDA experiment results. However, a significant ^1^O_2_ signal peak was found in the **WPP5-PBN-SR101** LHS, attributing to more energy of **WPP5-PBN** being transferred to excite **SR101** for ^1^O_2_ production (Figure 4d). According to reported studies [21,22], ^1^O_2_ is usually used as an oxidant for the photocatalytic generation of sulfoxide, which were significant for the chemical-pharmaceutical products. Due to the excellent ^1^O_2_ production ability, the **WPP5-PBN-SR101** LHS was used as the photocatalyst to oxidize the 4-methoxythioanisole to 1-methoxy-4-(methylsulfinyl)benzene, imitating the solar energy conversion to chemical energy of natural LHS (Scheme S1, Figures S15 and S16). As shown in Figure 4e,f, only 3% and 2% yields of the photooxidation products were observed in **WPP5-PBN** and **SR101** groups because of the poor production of ^1^O_2_. Notably, after the artificial light-harvesting process in **WPP5-PBN-SR101**, the harvested energy of the **WPP5-PBN** antenna was efficiently transferred to **SR101** for more ^1^O_2_ production, whose yield was significantly improved to 65%, displaying the **WPP5-PBN-SR101** LHS potential in artificial photosynthesis.

## 3. Materials and Methods

### 3.1. Synthesis of PBN

Compound **1** was synthesized according to our reported method [23].

Synthesis of Compound **2**: Compound **1** (1.17 g, 3.00 mmol), 1,4-phenylenediacetonitrile (0.23 g, 1.47 mmol), and tetrabutylammonium hydroxide (8 drop, 50%, TBAH) were added in 40 mL ethanol and stirred for 24 h. The solid was separated and washed with ethyl ether to afford Compound **2** (1.16 g, 1.29 mmol, 86%). ^1^H NMR (CDCl_3_, 400 MHz) δ (ppm): 8.24 (s, 2H), 8.13 (d, *J* = 8.8 Hz, 2H), 7.83–7.78 (m, 8H), 7.72 (s, 2H), 7.22 (dd, *J* = 8.8, 2.4 Hz, 2H), 7.15 (d, *J* = 2.0 Hz, 2H), 4.12 (t, *J* = 6.4 Hz, 4H), 3.43 (t, *J* = 6.8 Hz, 4H), 1.90–1.83 (m, 8H), 1.53–1.33 (m, 24H). ^13^C NMR (CDCl_3_, 100 MHz) δ (ppm): 158.95, 142.90, 135.94, 135.29, 130.95, 130.43, 128.80, 128.40, 127.55, 126.43, 125.77, 120.15, 118.27, 108.98, 106.57, 68.19, 34.12, 32.83, 29.47, 29.39, 29.36, 29.18, 28.77, 28.18, 26.09. HR-ESI-MS: *m*/*z* [M + H]^+^ calcd for [C_52_H_59_Br_2_N_2_O_2_]^+^ 901.2938, 903.2917, 904.2951, found 901.2936, 903.2893, 904.2961.

Synthesis of Compound **PBN**: Compound **2** (0.90 g, 1.00 mmol) and trimethylamine (5 mL, 2 M in THF) were added in 20 mL CHCl_3_ and refluxed overnight. The solvent was concentrated and washed with ethyl ether to afford Compound **PBN** (1.01 g, 0.99 mmol, 99%). ^1^H NMR (DMSO-*d*_6_, 400 MHz) δ (ppm): 8.40 (s, 2H), 8.28–8.26 (m, 2H), 8.16 (d, *J* = 8.8 Hz, 2H), 7.97–7.92 (m, 8H), 7.41 (s, 2H), 7.26 (d, *J* = 9.2 Hz, 2H), 4.15 (t, *J* = 6.4 Hz, 4H), 3.29–3.24 (m, 4H), 3.04 (s, 18H), 1.84–1.77 (m, 4H), 1.71–1.63 (m, 4H), 1.49–1.44 (m, 4H), 1.32–1.27 (m, 20H). ^13^C NMR (DMSO-*d*_6_, 100 MHz) δ (ppm): 158.83, 143.95, 135.96, 134.96, 131.39, 130.87, 129.36, 128.33, 127.88, 126.80, 125.83, 120.35, 118.60, 108.42, 107.32, 68.22, 65.74, 52.59, 29.39, 29.24, 29.08, 28.98, 26.23, 26.02, 22.50. HR-ESI-MS: m/*z* [M − 2Br]^2+^ calcd for [C_58_H_76_N_4_O_2_]^2+^ 430.2979, found 430.2973.

### 3.2. Construction of Supramolecular Nanoparticles

Firstly, the **PBN** solution in DMSO (0.02 M), **WPP5** solution in water (0.42 mM), and **SR101** solution in DMSO (0.20 mM) were prepared, respectively. Then, 10 μL **PBN** solution and 72 μL **WPP5** solution were mixed in water and diluted to 4 mL by ultrapure water for constructing **WPP5-PBN** nanoparticles. Next, 10 μL **PBN** solution, 72 μL **WPP5** solution, and (0–10) μL **SR101** solution were mixed in water and diluted to 4 mL by ultrapure water for constructing **WPP5-PBN-SR101** nanoparticles. The nanoparticles solution was prepared by ultrapure water under room temperature. The molar ratio of [**PBN**]:[**WPP5**]:[**SR101**] in artificial LHS for photocatalysis was 100:15:1.

### 3.3. Photooxidation of 4-Methoxythioanisole

4-Methoxythioanisole (80 μL) was added in the freshly prepared LHS solution (4 mL) in a 10 mL glass vial. The mixture was bubbled by air, and then irradiated by 9 W 365 nm LED at room temperature to obtain the photooxidation product.

## 4. Conclusions

In summary, we have successfully constructed a new artificial light-harvesting system (LHS) by the supramolecular interaction of water-soluble phosphate-pillar[5]arene (**WPP5**) host molecule and phenyl-bis-naphthyl derivative (**PBN**) guest molecule. Based on the aggregate emission mechanism in the AIE effect, **WPP5-PBN** nanoparticles emitted a significant yellow fluorescence after the self-assembly process, displaying prospective donor ability in artificial LHSs. Due to **WPP5-PBN**’s yellow fluorescence fully covering the absorption of sulforhodamine 101 (**SR101**, acceptor), **WPP5-PBN** conducted as a light-harvesting antenna and transferred energy to **SR101** after loading **SR101** in **WPP5-PBN** nanoparticles, whose energy transfer efficiency and antenna effect were 66.32% and 22.34. Benefitting from the efficient energy transfer process in the **WPP5-PBN-SR101** LHS, more energy was transferred to excite **SR101** and produce a significant singlet oxygen (^1^O_2_), which had potential for the photooxidation reaction. Subsequently, the **WPP5-PBN-SR101** LHS was used as a photocatalyst to oxidize the 4-methoxythioanisole to 1-methoxy-4-(methylsulfinyl)benzene, whose yield was significantly improved to 65%, imitating the solar energy conversion to chemical energy.

## Data Availability

The original contributions presented in this study are included in the article and Supplementary Materials. Further inquiries can be directed to the corresponding author.

## Supplementary Materials

The following supporting information can be downloaded at: https://www.mdpi.com/article/10.3390/molecules30224424/s1. Figure S1. ^1^H NMR spectrum (400 MHz, CDCl_3_) of Compound **1**. Figure S2. ^1^H NMR spectrum (400 MHz, CDCl_3_) of Compound **2**. Figure S3. ^13^C NMR spectrum (100 MHz, CDCl_3_) of Compound **2**. Figure S4. HR-ESI-MS spectrum of Compound **2**. Figure S5. ^1^H NMR spectrum (400 MHz, DMSO-*d*_6_, 298 K) of **PBN**. Figure S6. ^13^C NMR spectrum (100 MHz, DMSO-*d*_6_, 298 K) of **PBN**. Figure S7. HR-ESI-MS spectrum of **PBN**. Figure S8. The XPS spectrum of **WPP5-PBN-SR101**. Figure S9. Fluorescence lifetime of **WPP5-PBN** nanoparticles. Figure S10. Fluorescence lifetime of **WPP5-PBN-SR101** nanoparticles. Figure S11. Fluorescence spectra of **WPP5-PBN** and **WPP5-PBN-SR101** under 365 nm excitation. Figure S12. Fluorescence spectrum of **WPP5-PBN** (black line), which was normalized with **WPP5-PBN-SR101** (red line) at 551 nm; Fluorescence spectra of **WPP5-PBN-SR101** (red line under 365 nm excitation and blue line under 535 nm excitation). Figure S13. The ^1^O_2_ oxidation mechanism of ABDA. Figure S14. The UV–Vis absorbance spectra of (a) free ABDA, (b) ABDA+**WPP5-PBN**, (c) ABDA+**SR101**, and (d) ABDA+**WPP5-PBN-SR101** after 365 nm irradiation (9 W) for different times. Scheme S1. The photooxidation reaction. Figure S15. ^1^H NMR spectrum (400 MHz, CDCl_3_, 298 K) of photooxidation product. Figure S16. HR-ESI-MS spectrum of photooxidation product. Reference [24] is cited in the supplementary materials.
