# Supplementary material for: Phenyl-Bis-Naphthyl Derivative-Based Artificial Light-Harvesting System for Singlet Oxygen Oxidation
Source: Molecules. 2025 Nov 16;30(22):4424. doi: 10.3390/molecules30224424 (PMC12655598; doi:10.3390/molecules30224424)
Supplement: Supplementary file 1 [file molecules-30-04424-s001.zip › molecules-3944005-supplementary.pdf]

Supporting Information

Phenyl-Bis-Naphthyl Derivative-Based Artificial Light-Harvesting System for Singlet Oxygen Oxidation

Liangtao Pu [1], Yonglei Chen [1] and Guangping Sun [2,*]

[1] School of Urban Construction, Changzhou University, Changzhou 213164, China
[2] School of Chemistry and Chemical Engineering, Nantong University, Nantong 226019, China
* Correspondence: sunguangping1989@ntu.edu.cn

Table of Contents

1. General information 2

2. Synthesis of guest molecule PBN 2

3. The XPS investigation of WPP5-PBN-SR101 6

4. Lifetime of supramolecular nanoparticles 7

5. Energy transfer efficiency 7

6. Antenna effect calculation 8

7. Determination of $^1O_2$ in WPP5-PBN-SR101 9

8. Investigation of the photooxidation reaction 9

9. References 11

1. General information

All reactions were performed in air atmosphere unless otherwise stated. The commercially available reagents and solvents were either employed as purchased or dried according to procedures described in the literatures. Column chromatography was performed with silica gel (200 – 300 mesh) produced by Qingdao Marine Chemical Factory, Qingdao (China). All yields were given as isolated yields. NMR spectra were recorded on a Bruker 400 MHz spectrometer with internal standard tetramethylsilane (TMS) and solvent signals as internal references at room temperature, and the chemical shifts (δ) were expressed in ppm and *J* values were given in Hz. High-resolution electrospray ionization mass spectra (HR-ESI-MS) were recorded on Agilent 6540Q-TOF LCMS and Q-TOF (AB SCIEX X500R) equipped with an electrospray ionization (ESI) probe operating in the positive-ion mode with direct infusion. Dynamic light scattering (DLS) was carried out on a computer-controlled laser diffraction apparatus (Zetasizer Nano-ZS90, Malvern Instruments Ltd., Worcestershire, UK). The UV-Vis absorption spectra were measured on a Perkin Elmer Lambda 35 UV-Vis Spectrometer. The emission spectra were recorded on a Hitachi F-7000 Fluorescence Spectrometer. Transmission electron microscope (TEM) investigations were carried out on a Talos F200X instrument. The fluorescence lifetimes were measured employing time correlated single photon counting on a FS5 instrument (Edinburg Instruments Ltd., Livingstone, UK). The $^1O_2$ was measured by Bruker Magnettech ESR5000. X-ray photoelectron spectroscopy (XPS) was measured by Thermo K-Alpha.

2. Synthesis of guest molecule PBN

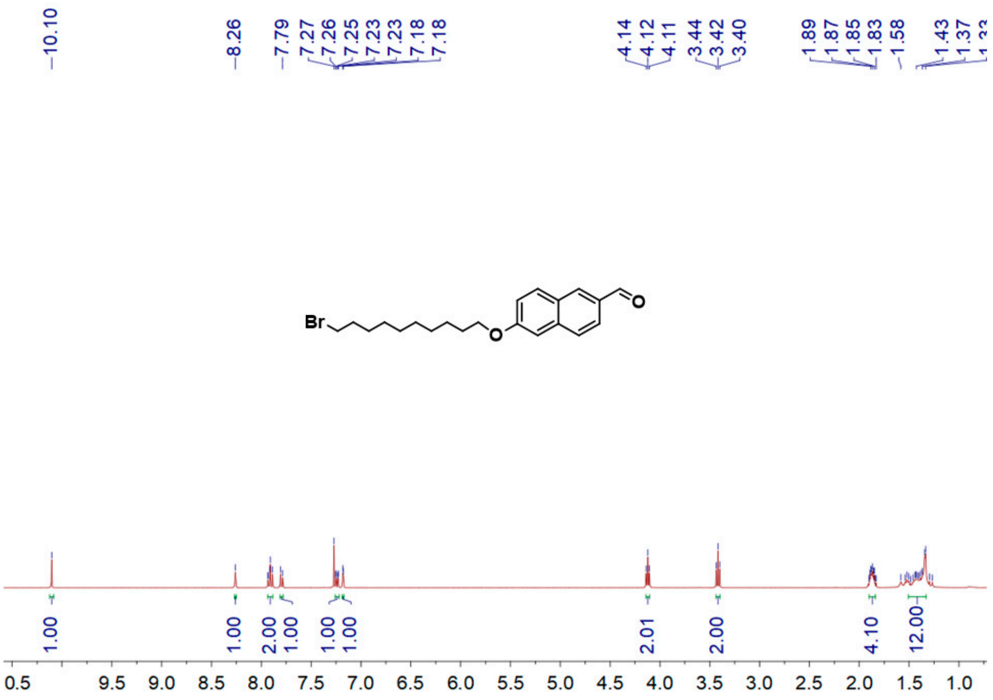

Figure S1. ^{1}H NMR spectrum (400 MHz, $CDCl_3$) of compound **1**.

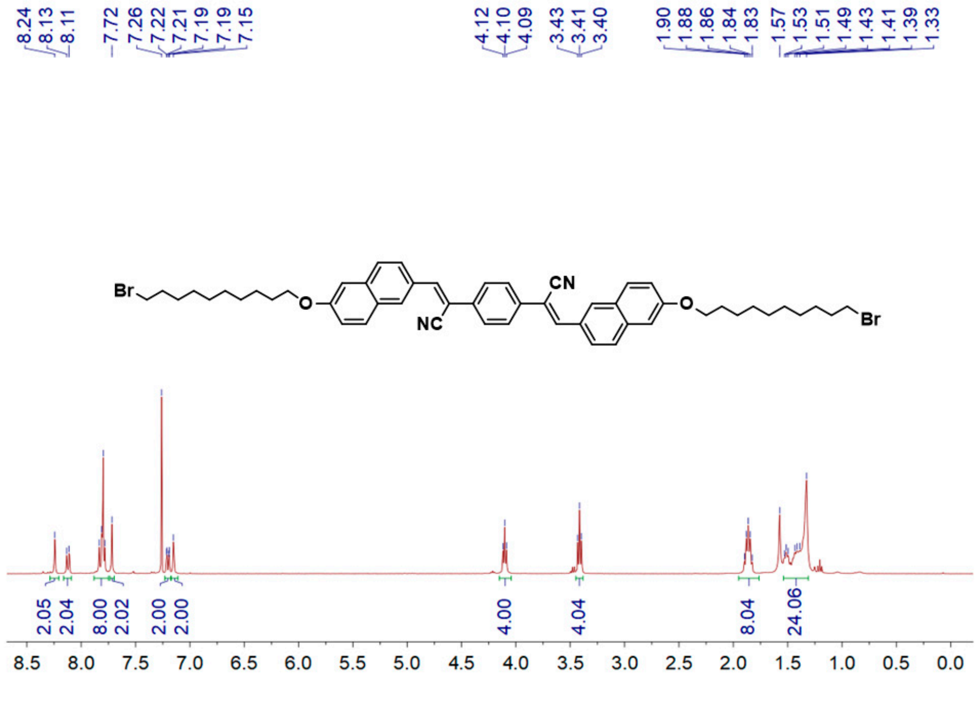

Figure S2. ^{1}H NMR spectrum (400 MHz, $CDCl_3$) of compound **2**.

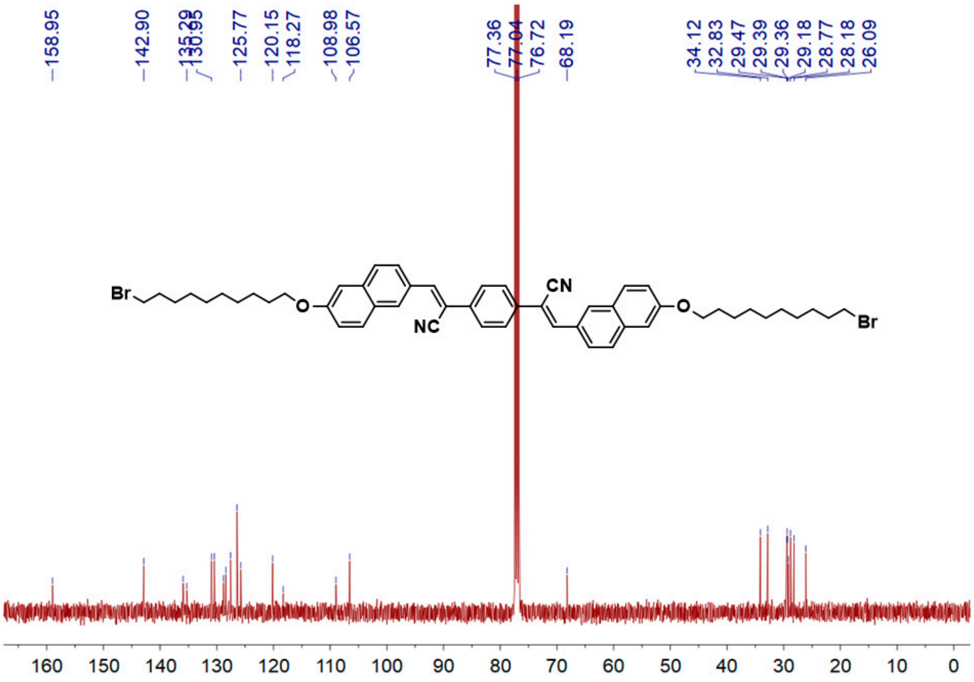

Figure S3. ^{13}C NMR spectrum (100 MHz, $CDCl_3$) of compound **2**.

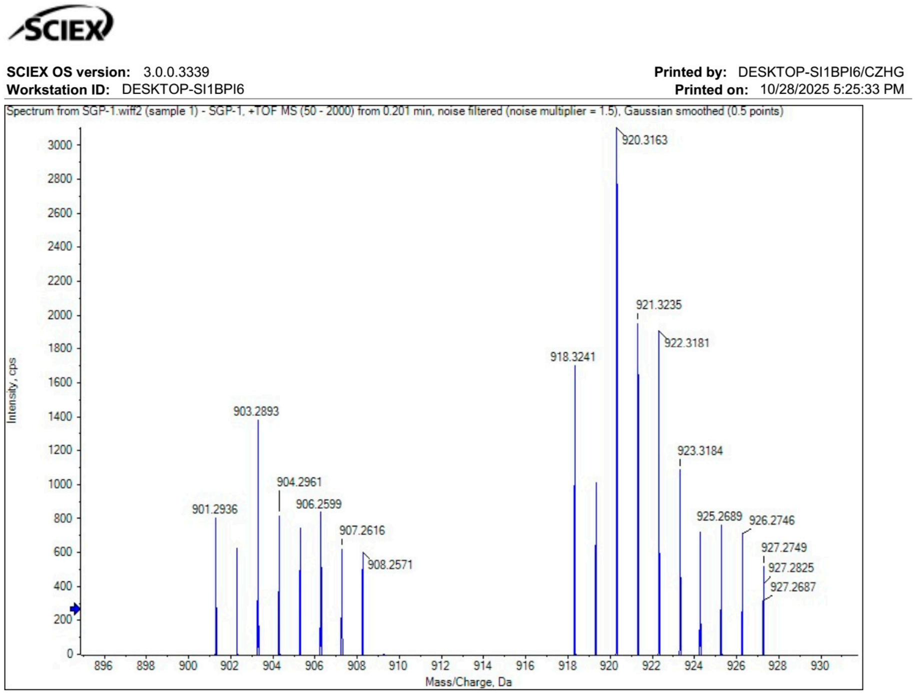

Figure S4. HR-ESI-MS spectrum of compound **2**.

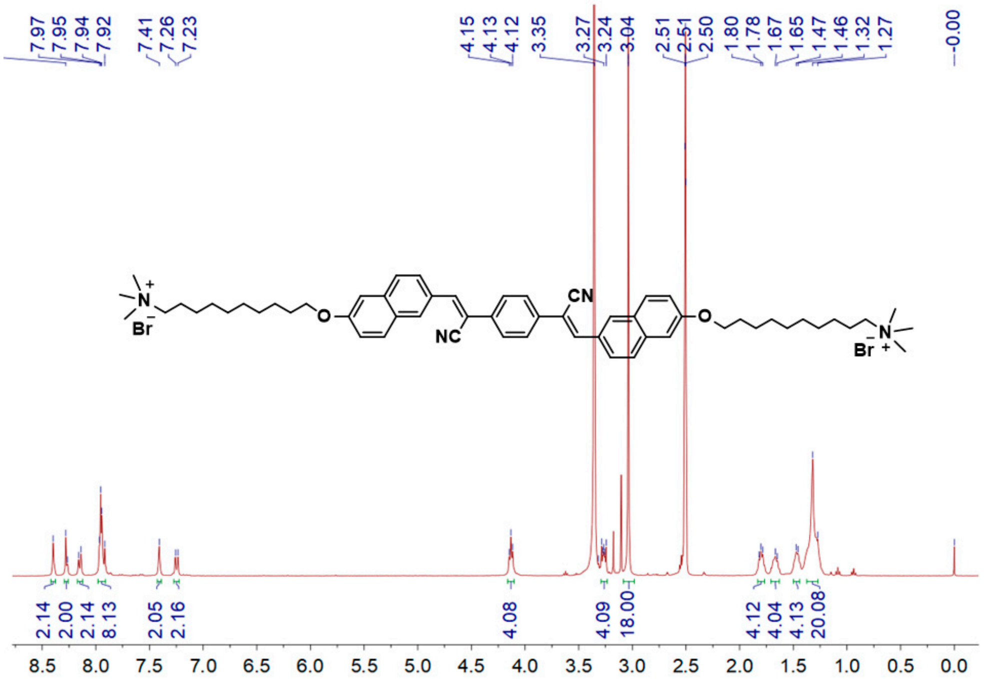

Figure S5. ^{1}H NMR spectrum (400 MHz, DMSO-d_6, 298 K) of **PBN**.

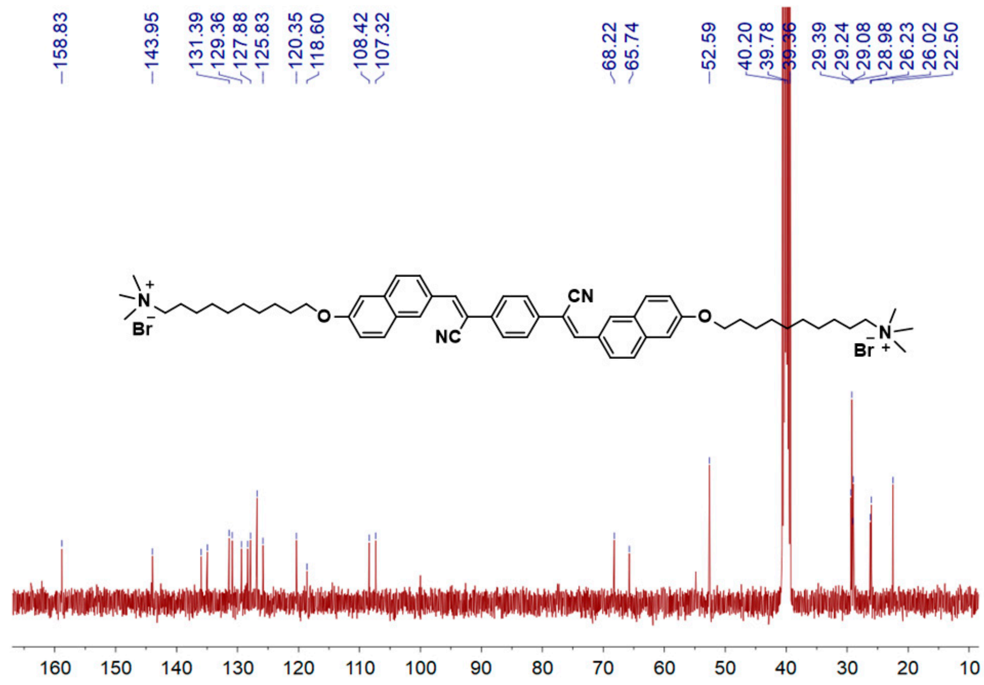

Figure S6. ^{13}C NMR spectrum (100 MHz, DMSO-d_6, 298 K) of **PBN**.

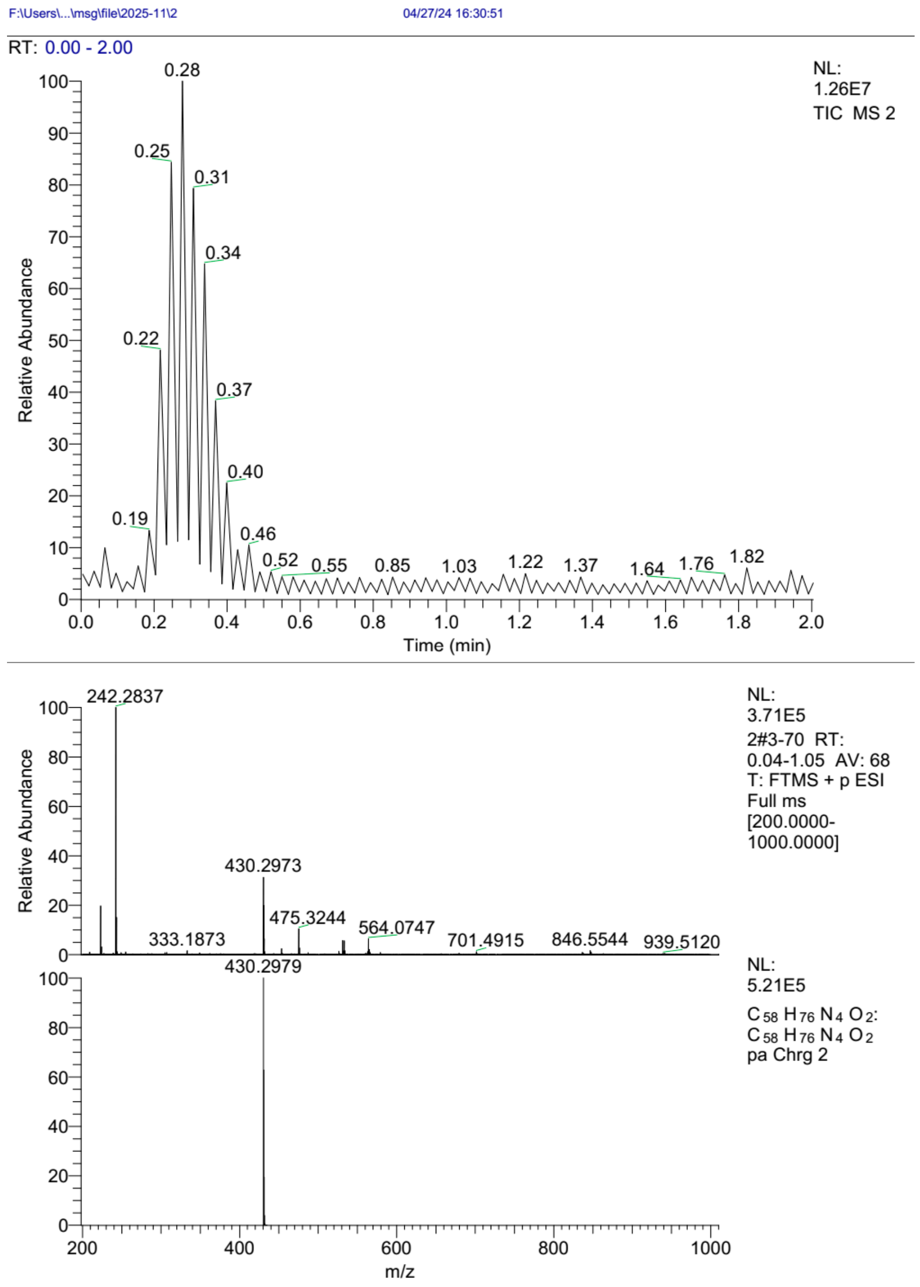

Figure S7. HR-ESI-MS spectrum of **PBN**.

3. The XPS investigation of WPP5-PBN-SR101

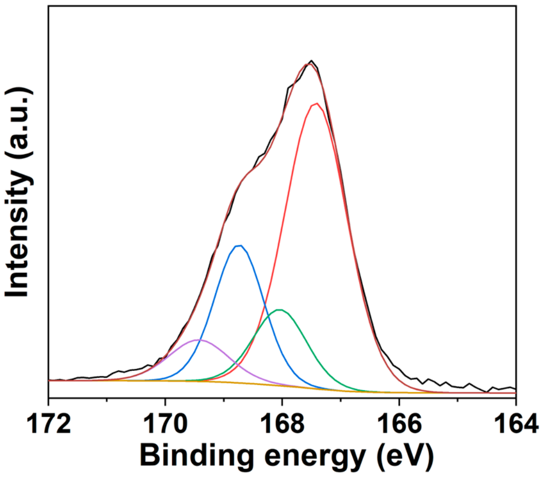

Figure S8. The XPS spectrum of **WPP5-PBN-SR101**.

4. Lifetime of supramolecular nanoparticles

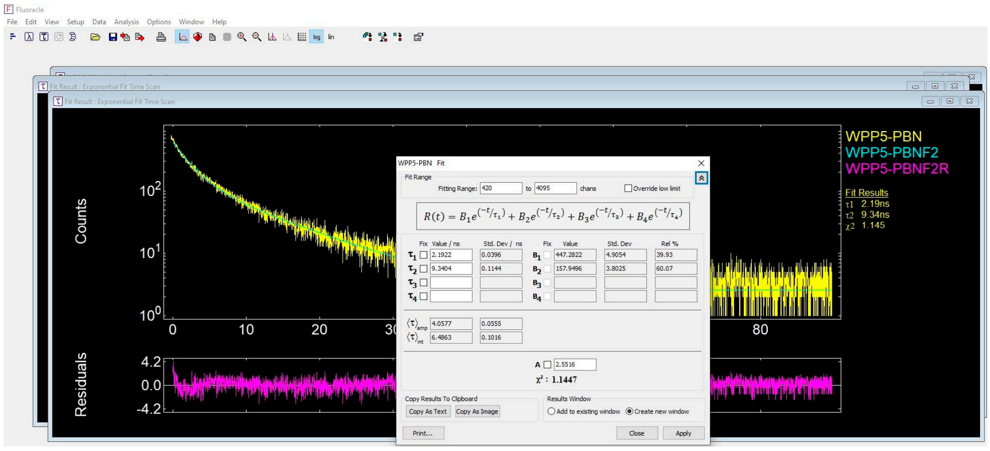

Figure S9. Fluorescence lifetime of **WPP5-PBN** nanoparticles.

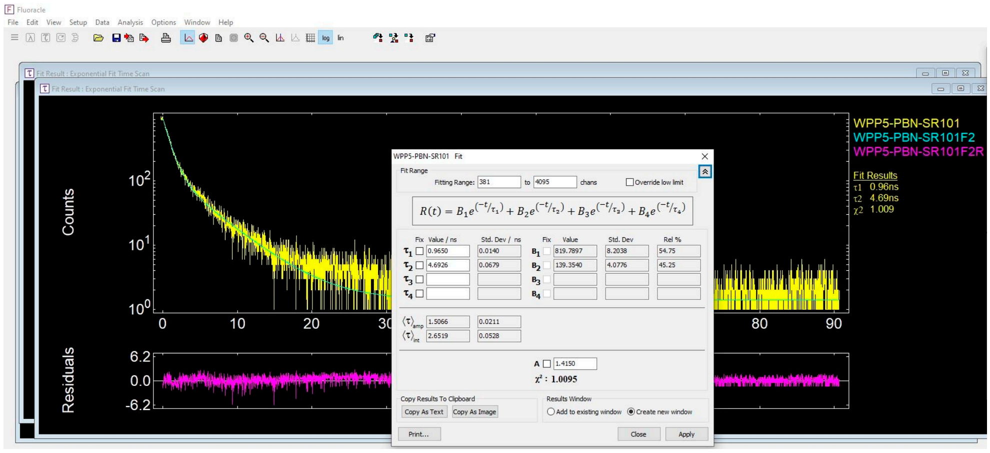

Figure S10. Fluorescence lifetime of **WPP5-PBN-SR101** nanoparticles.

5. Energy transfer efficiency

The energy transfer efficiency (Φ_{ET}) was calculated according to the equation of "Φ_{ET} = 1 – I_{DA}

/ I_D", where I_{DA} and I_D are the fluorescence intensities of **WPP5-PBN-SR101** and **WPP5-PBN** at 551 nm under 365 nm excitation.[S1,S2]

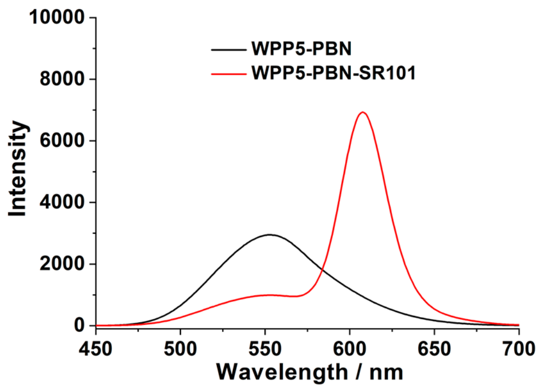

Figure S11. Fluorescence spectra of **WPP5-PBN** and **WPP5-PBN-SR101** under 365 nm excitation, [**PBN**] = 50.00 μM, [**WPP5**] = 7.50 μM, and [**SR101**] = 0.50 μM.

According to the intensity of supramolecular nanoparticles at 551 nm, the energy transfer efficiency (Φ_{ET}) of **WPP5-PBN-SR101** was calculated to be 66.32% in water.

6. Antenna effect calculation

The antenna effect (AE) was calculated according to the equation of "AE = ($I_{DA,365} - I_{D,365}$) / $I_{DA,535}$", where $I_{DA,365}$ and $I_{DA,535}$ are the fluorescence intensities of **WPP5-PBN-SR101** at 608 nm with the excitation of the donor (**PBN**) at 365 nm and the direct excitation of the acceptor (**SR101**) at 535 nm, respectively. $I_{D,365}$ is the fluorescence intensity of **WPP5-PBN** at 608 nm, which was normalized with **WPP5-PBN-SR101** at 551 nm.[S1,S2]

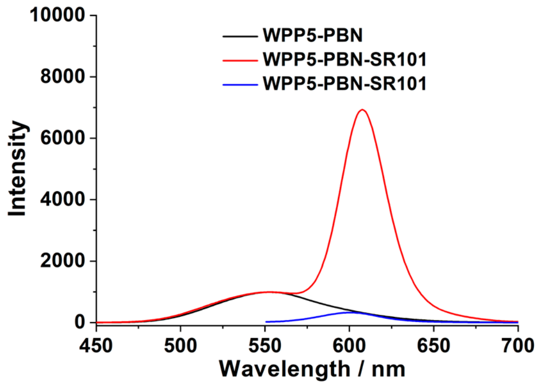

Figure S12. The normalized fluorescence spectrum of **WPP5-PBN** (black line) according to the intensity of **WPP5-PBN-SR101** (red line) at 551 nm and the fluorescence spectra of **WPP5-PBN-SR101** (red line was under 365 nm excitation and blue line was under 535 nm excitation), [**PBN**] = 50.00 μM, [**WPP5**] = 7.50 μM, and [**SR101**] = 0.50 μM.

According to the intensity of supramolecular nanoparticles at 608 nm, the antenna effect of **WPP5-PBN-SR101** was calculated to be 22.34 in water.

7. Determination of $^1O_2$ in WPP5-PBN-SR101

The $^1O_2$ was detected by using 9,10-anthracenediyl-bis(methylene)dimalonic acid (ABDA) as the UV-Vis absorbance probe.

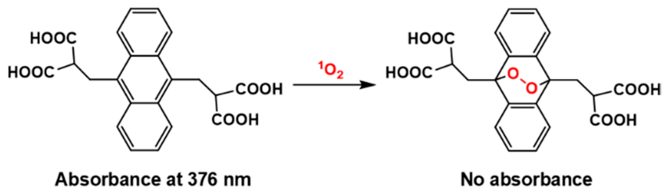

Figure S13. The $^1O_2$ oxidation mechanism of ABDA.

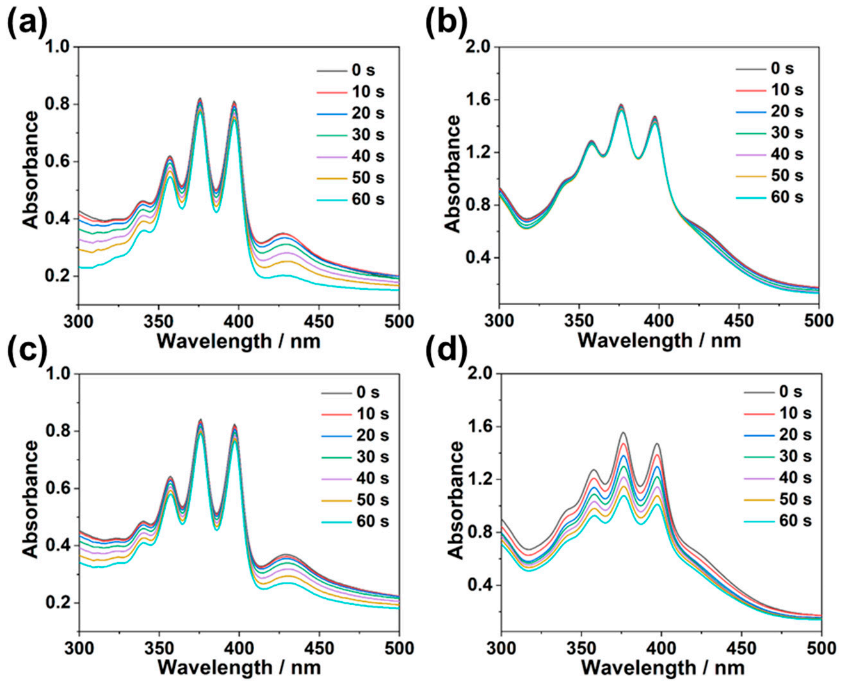

Figure S14. The UV-Vis absorbance spectra of (a) free ABDA, (b) ABDA+**WPP5-PBN**, (c) ABDA+**SR101**, and (d) ABDA+**WPP5-PBN-SR101** after 365 nm irradiation (9 W) for different times.

8. Investigation of the photooxidation reaction

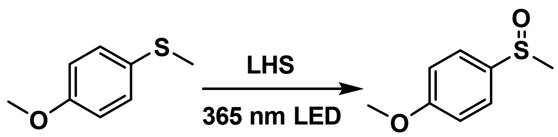

Scheme S1. The photooxidation reaction.

According to previous literatures,[S3,S4] 80 μL 4-methoxythioanisole was added in the freshly prepared LHS solution (4 mL) in a 10 mL glass vial. The mixture was bubbled by air, and then irradiated by 9 W 365 nm LED at room temperature for corresponding time. The distance between the light and reaction vessel was 20 cm. After that, the mixture was extracted with 20 mL ethyl acetate and the combined organic layer was purified by column chromatography (petroleum ether/ethyl acetate = 5:1, v/v) to afford photooxidation product. ^{1}H NMR and HR-ESI-MS spectra of photooxidation product was successfully obtained. ^{1}H NMR (400 MHz, $CDCl_3$, 298 K) δ (ppm): 7.61 (d, J = 8.8 Hz, 2H), 7.05 (d, J = 8.8 Hz, 2H), 3.86 (s, 3H), 2.71 (s, 3H). HR-ESI-MS: m/z $[M + H]^+$ calcd for $[C_8H_{11}O_2S]^+$ 171.0474, found 171.0473.

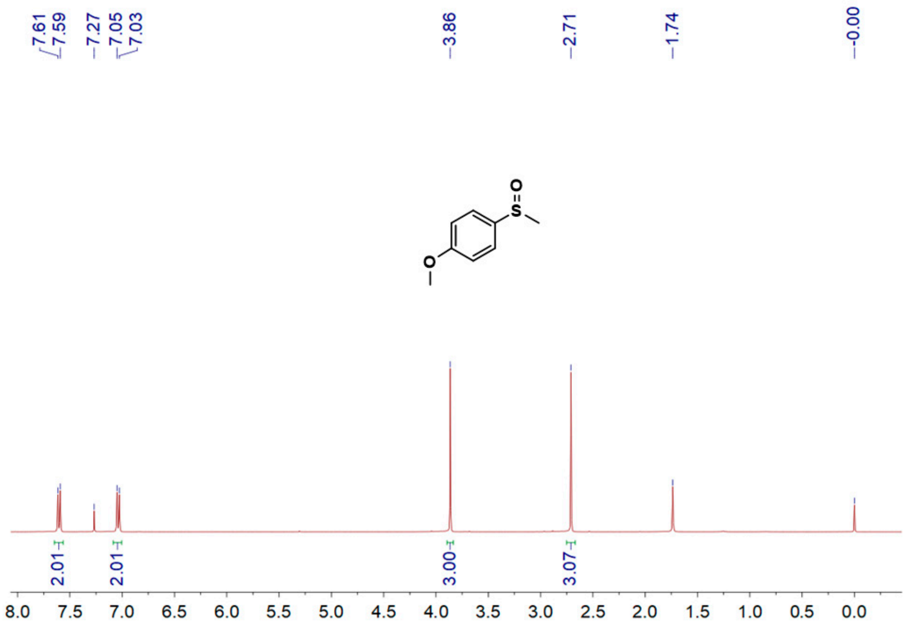

Figure S15. ^{1}H NMR spectrum (400 MHz, $CDCl_3$, 298 K) of photooxidation product.

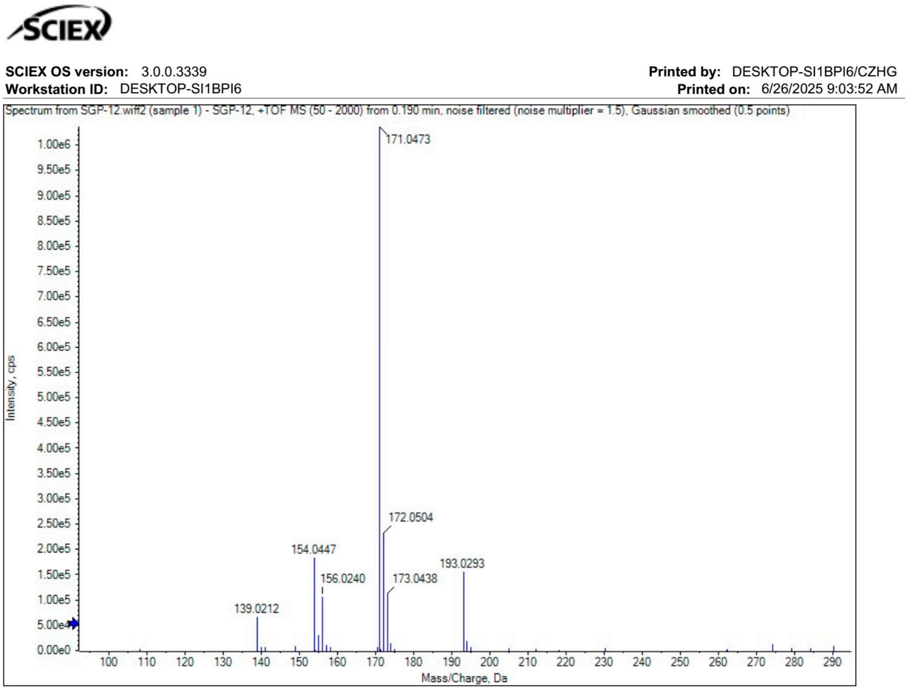

Figure S16. HR-ESI-MS spectrum of photooxidation product.

9. References

S1. Sun, G.; Li, M.; Cai, L.; Zhu, J.; Tang, Y.; Yao, Y.; Carbazole-based artificial light-harvesting system for photocatalytic cross-coupling dehydrogenation reaction. *Chem. Commun.* **2024**, *60*, 1412-1415.

S2. Sun, G.; Cai, L.; Cui, H.; Hu, Y.; Wang, J.; Wang, M.; Zhu, J.; Sun T.; Tang, Y.; Naphthalenyl-phenylacrylonitrile-based supramolecular aqueous artificial light-harvesting system for photochemical catalysis. *Dyes Pigments* **2022**, *201*, 110257.

S3. Sun, G.; Li, M.; Li, J.; Feng, J.; Yan, Z.; Sun, Y.; Pu, L.; Zhu, J.; Tang, Y.; Yao, Y.; Enhanced emission in a supramolecular artificial light-harvesting system for a photocatalytic thiol-ene reaction. *Chem. Commun.* **2025**, *61*, 6360-6363.

S4. Li, X.-L.; Cheng, D.-L.; Niu, K.-K.; Liu, H.; Yu, S.-S.; Wang, Y.-B.; Xing, L.-B.; Construction of supramolecular dimer photosensitizers based on triphenylamine derivatives and cucurbit[8]uril for photocatalysis. *J. Mater. Chem. A* **2023**, *11*, 24911-24917.
